# Water Formation Reaction under Interfacial Confinement: Al_0.25_Si_0.75_O_2_ on O-Ru(0001)

**DOI:** 10.3390/nano12020183

**Published:** 2022-01-06

**Authors:** Jorge Cored, Mengen Wang, Nusnin Akter, Zubin Darbari, Yixin Xu, Burcu Karagoz, Iradwikanari Waluyo, Adrian Hunt, Dario Stacchiola, Ashley Rose Head, Patricia Concepcion, Deyu Lu, Jorge Anibal Boscoboinik

**Affiliations:** 1 Instituto de Tecnología Química, Universitat Politècnica de València-Consejo Superior de Investigaciones Científicas (UPV-CSIC), Avenida de los Naranjos s/n, 46022 Valencia, Spain; jorcoban@upvnet.upv.es (J.C.); pconcepc@upvnet.upv.es (P.C.); 2 Center for Functional Nanomaterials, Brookhaven National Laboratory, Upton, NY 11973, USA; mwang.mse@gmail.com (M.W.); nusninakter@gmail.com (N.A.); zubin.darbari@stonybrook.edu (Z.D.); yixin.xu@stonybrook.edu (Y.X.); bkaragoz@bnl.gov (B.K.); djs@bnl.gov (D.S.); ahead@bnl.gov (A.R.H.); 3 Materials Science and Chemical Engineering Department, Stony Brook University, Stony Brook, NY 11790, USA; 4 National Synchrotron Light Source II, Brookhaven National Laboratory, Upton, NY 11973, USA; iwaluyo@bnl.gov (I.W.); adhunt@bnl.gov (A.H.)

**Keywords:** water formation reaction, ambient pressure X-ray photoelectron spectroscopy, density functional theory, aluminosilicate bilayer film, reaction pathway, interfacial confinement, nanoreactor

## Abstract

Confined nanosized spaces at the interface between a metal and a seemingly inert material, such as a silicate, have recently been shown to influence the chemistry at the metal surface. In prior work, we observed that a bilayer (BL) silica on Ru(0001) can change the reaction pathway of the water formation reaction (WFR) near room temperature when compared to the bare metal. In this work, we looked at the effect of doping the silicate with Al, resulting in a stoichiometry of Al_0.25_Si_0.75_O_2_. We investigated the kinetics of WFR at elevated H_2_ pressures and various temperatures under interfacial confinement using ambient pressure X-ray photoelectron spectroscopy. The apparent activation energy was lower than that on bare Ru(0001) but higher than that on the BL-silica/Ru(0001). The apparent reaction order with respect to H_2_ was also determined. The increased residence time of water at the surface, resulting from the presence of the BL-aluminosilicate (and its subsequent electrostatic stabilization), favors the so-called disproportionation reaction pathway (*H_2_O + *O ↔ 2 *OH), but with a higher energy barrier than for pure BL-silica.

## 1. Introduction

The effects of nanoscale confinement are common in nature, and their importance is becoming increasingly recognized in different chemical research areas [1,2]. Depending on the size and shape of the confined space, the “molecule-host material” or “molecule-molecule” interactions can be altered or even controlled, which can have a fundamental impact in a variety of fields, especially in catalysis [3,4,5,6]. Some ordered nanoporous materials, such as zeolites or metal-organic frameworks (MOF), have been used to explore confinement effects in heterogeneous catalysis. In addition to the sort of interaction of molecules with the material, the extent of the confinement can affect the activity and the selectivity of a chemical process, introducing steric requirements for substrates participating in the reaction [7]. For instance, carbon-derived materials (such as carbon nanotubes, CNTs) have been used to drive reactions with improved formation rates, favoring the activation of stable chemical functionalities, such as C–H bonds [8,9]. Moreover, the combination of CNTs with metallic nanoparticles (i.e., copper) has been shown as a good strategy to improve the catalytic performance in the hydrogenation of methyl acetate to methanol and ethanol. In that work, the selectivity to the C_2_ alcohol was found to be dependent on the inner diameter of the CNT, being the nano-confinement also responsible for the improved long-term stability of the catalyst [10]. On the other hand, zeolitic materials have been applied in a wide variety of industrial processes (Fischer–Tropsch, partial oxidation of aromatic molecules, C–C coupling, etc.) because of their structural versatility and outstanding thermal and chemical stabilities [11].

Another type of architecture that attracted growing attention in past decades is the confined space that appears in weakly bound composites formed by a metal substrate and a thin film or layered 2D structure. In this sense, the interfacial confinement existing in these materials can also alter the mechanism operating in a particular chemical process [12,13]. For example, it is well known that carbonaceous deposits formed during hydrogenation reactions involving carbon-based compounds can poison metallic surfaces, blocking active sites. However, the adequate use of 2D-graphene covering a Pt(111) surface creates a unique confined interface that reduces the activation energy for the CO oxidation reaction by 0.15 eV, compared to a bare platinum surface [14]. Furthermore, it is possible to promote the hydrogen evolution reaction (HER) on a nickel surface by depositing graphene. As a consequence, the initial dissociative adsorption of H_2_ molecules at the metal/graphene interface is ~0.2 eV weaker compared to the bare Ni. Other side phenomena taking place during HER can be enhanced due to this confinement, such as H_2_ spillover, to increase the reaction rate [15].

Additionally, porous thin-film silicates weakly interacting with metallic supports (via van der Waals forces, vdW) have been applied for the same purpose. These materials, considered 2D models (or simplified mimics) of zeolitic structures, consist of a ~0.5 nm thick bilayer SiO_2_ scaffold of hexagonal prims. The parallel –(Si–O–Si)– sheets that form the bilayer are interconnected by oxygen atoms, generating pores of about 5 Å [16,17]. Different crystallinities can be obtained depending on the synthetic procedure, including vitreous [18] or mixed vitreous-crystalline arrangements [19,20]. The structure of these composites can be characterized using surface science tools and theoretical approaches [12,21,22,23].

Due to the crystalline porous structure of 2D bilayer silica, the permeation of small molecules or atoms (such as CO, O_2_, H_2_, H_2_O, Ar, Au, Pd) through the 2D nanospace is feasible, permitting in this manner the interaction of these adsorbates with the bilayer or with the metallic surface [24,25,26,27,28,29,30]. Furthermore, the structural and electronic features of these SiO_2_/metal heterojunctions (and subsequently, the nature of the interfacial space that is created) can be tuned by modifying the surface where the silicate is grown (e.g., Pd(111) and (100) [31,32], Pt(111) [33] and Ru(0001) [16,21]). Additionally, it is possible to control the magnitude of the interfacial distance by replacing some of the Si atoms with Al during the synthesis [34] or by introducing chemisorbed species into the nano-space [35], inducing electrostatic interactions. Some examples of reactions affected by this confinement, such as CO oxidation [17] or furfuryl alcohol evolution to different furan derivatives [36], have been recently published by our group, highlighting the importance of exploring this novel chemistry at a subnanometric scale [24].

**Figure 1:**
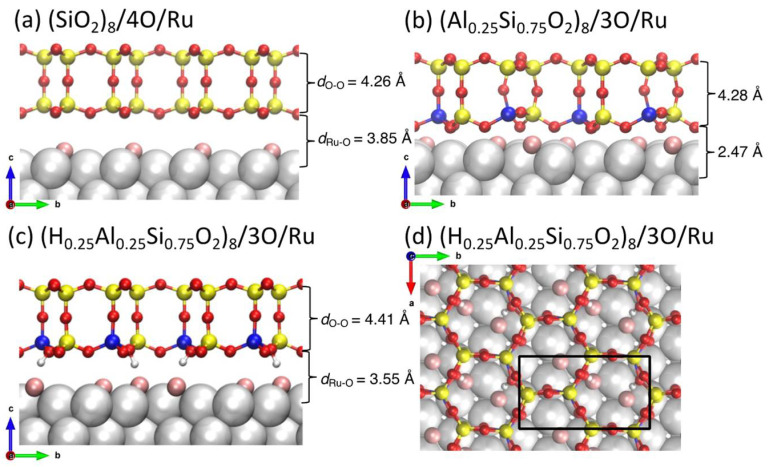
Atomic structures of (**a**) (SiO_2_)_8_/4O/Ru and (**b**) (Al_0.25_Si_0.75_O_2_)_8_/3O/Ru(0001). Side (**c**) and top (**d**) views of the bilayer aluminosilicate film growth on Ru(0001) with two H^+^ bound to the bridging O in (Al–O^−^)–Si to compensate the framework charge [i.e., (H_0.25_Al_0.25_Si_0.75_O_2_)_8_/3O/Ru(0001)]. The black rectangle on the top view (**d**) indicates the unit cell. Color code: Ru (silver), Si (yellow), Al (blue), H (white), O in aluminosilicate (red), and O chemisorbed on Ru (pink).

The structures that will be the subject of study in this work are presented in Figure 1. Side views of bilayer silica (Figure 1a), bilayer aluminosilicate (Figure 1b), and hydroxylated bilayer aluminosilicate (Figure 1c) supported on Ru(0001) are shown, together with the top view of the hydroxylated aluminosilicate bilayer (Figure 1d). All of them are based on density functional theory (DFT) calculations described in more detail below.

On the other hand, the chemical process selected to carry out this study is the water formation reaction (WFR). Despite its apparent simplicity, the mechanism of WFR has been the object of investigation because the individual steps involved in the reaction pathway (i.e., dissociative adsorption of H_2_ or O_2_ molecules onto a metal, or the combination of *H and *O to yield the hydroxyl intermediate) are common to very diverse catalytic transformations. For instance, the oxidation of fuel molecules (H_2_) is a key electrochemical process that takes place in solid oxide fuel cells (SOFC) [37]. Some of the aforementioned steps also occur in hydrogen or oxygen evolution reactions (HER and OER, respectively) [38]. Therefore, significant efforts have been made to understand the fundamentals of this process, in order to discover new active centers that compete with platinum-based materials [39].

**Figure 2:**
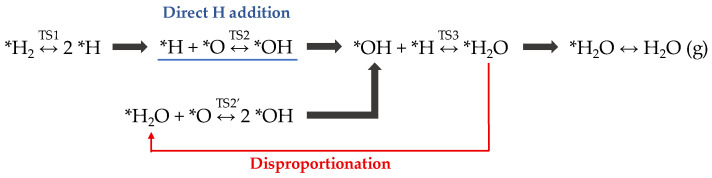
Dual-path reaction mechanism of water formation reaction (WFR) reported on the Pt(111) surface. * indicates the species adsorbed on the platinum surface.

In fact, the WFR has been studied on the Pt(111) surface over a wide temperature range: i.e., below 150 K [40,41,42] and 250–450 K [43,44,45]. Based on DFT studies [46], combined with high-resolution electron energy loss spectroscopy (HREELS) and scanning tunneling microscope (STM) experiments [47], Figure 2 shows the dual-path mechanism suggested for the WFR on Pt(111) substrate.

The WFR mechanism starts with the H_2_ molecule adsorption and dissociation onto the metallic surface (TS1). Afterward, the rate-limiting step, i.e., hydroxyl group formation, occurs. As marked in Figure 2, *OH intermediate formation can be produced via two alternative pathways: (a) a direct hydrogen addition (*H + *O ↔ *OH; TS2, blue line) or (b) a disproportionation pathway (*H_2_O + *O ↔ 2 *OH; TS2′, red arrow) [47]. In the latter option, an *H_2_O molecule that has already been synthesized combines with a chemisorbed oxygen to form two *OH. The “activation” of one or the other pathway depends on both the reaction temperature and the desorption temperature of water on the material. For Pt(111), this temperature is found around 150 K. Then, below that point, the disproportionation pathway (TS2′) dominates, with a low activation energy of ~0.33 eV [46]. However, above the water desorption temperature, *OH formation occurs via direct H addition (TS2), exhibiting higher activation energy (~1 eV) [46].

Besides platinum, ruthenium can also catalyze the WFR [48,49]. Hence, since bilayer silicates and aluminosilicates are well studied on Ru(0001), this architecture lends itself as an ideal model to study the effect of interfacial confinement in the reactivity of this surface. In this system, O_2_ molecules can permeate through the silicate and chemisorb dissociatively on the Ru(0001) surface. There, *O can be reduced by H_2_ under mild conditions to generate water, which finally desorbs from the interface as a product [34,35,50,51]. In prior work by our group (using AP-XPS) and by Prieto et al. (using LEEM), the WFR was studied under confinement at the BL-silica/Ru(0001), reporting a comparable decrease of the apparent activation energy (E^app^) by 0.38 eV and 0.32 eV, respectively, with respect to the Ru(0001) case [52,53]. Moreover, temperature-programmed desorption (TPD) experiments and high-resolution electron energy loss spectroscopy (HREELS) characterization demonstrated that the dual-path WFR mechanism proposed for Pt(111) in Figure 2 also operates on bare Ru(0001) [48,49]. Finally, a detailed study of the kinetic aspects of confinement aiming at understanding the distribution of species across the reaction fronts and the differences in the E^app^ have been recently reported by Prieto et al. [54]. In that work, carried out at 540 K and low H_2_ pressure (~10^−7^ Torr) on a crystalline BL-SiO_2_/Ru(0001) sample, they reported that the H-adsorption step is strongly affected by the presence of the silica bilayer, influencing the propagation of the reaction cascade.

In the present study, synchrotron-based ambient pressure X-ray photoelectron spectroscopy (AP-XPS) was used to determine the E^app^ of the WFR through the reduction of chemisorbed oxygen at elevated H_2_ pressures on the BL-aluminosilicate/Ru(0001) interface. The reaction order with respect to H_2_ was also determined experimentally. Moreover, DFT was used to examine the WFR at this confined interface, considering both discussed reaction pathways to produce the *OH intermediate, namely: the direct hydrogenation (TS2) and the disproportionation (TS2′). The energy profiles for both alternatives were compared to understand the effect of doping the silicate with Al in the WFR under confinement.

## 2. Materials and Methods

### 2.1. Material Synthesis

The Ru(0001) single crystal surface was cleaned with several cycles of Ar^+^ sputtering and annealing at 1200 K (e-beam heating). The temperature was measured by a K-type thermocouple attached to the side of the sample. The surface was then exposed to 3 × 10^−6^ Torr O_2_ at 1200 K in order to form a (2 × 2)-3O/Ru(0001) surface. The aluminosilicate bilayer film was grown on the (2 × 2)-3O/Ru(0001) surface as described in detail elsewhere [21]. Briefly, Si and Al were thermally evaporated onto the (2 × 2)-3O/Ru(0001) surface at room temperature under 2 × 10^−7^ Torr O_2_, followed by oxidation at 1200 K in 3 × 10^−6^ Torr O_2_ for 10 min. Then, the temperature was decreased to 300 K, keeping the O_2_ pressure constant. The bilayer nature of the aluminosilicate was verified using infrared reflection absorption spectroscopy (IRRAS). The IRRAS system is home-built, using a Bruker Vertex 80V spectrometer (Bruker, Rosenheim, Germany). After synthesis, the sample was transported through air to the ambient pressure (AP)-XPS system at the IOS beamline of the National Synchrotron Light Source II (NSLS-II, Upton, NY, USA). The AP-XPS system is home-built using a Phoibos NAP150 from SPECS GmbH, Berlin, Germany. To clean the surface from airborne carbonaceous contamination, the surface was firstly annealed to 700 K in 5 × 10^−2^ O_2_, followed by annealing to 373 K in 1 Torr of H_2_. The sample cleaning procedure was monitored in situ by AP-XPS. The annealing in H_2_ was performed to remove the chemisorbed oxygen formed during the previous step [50].

### 2.2. Computational Methods

DFT calculations were performed using the projector augmented wave method implemented in the Vienna Ab initio simulation package (VASP) [55,56]. The non-local vdW interactions were described by the optB86b-vdW functional [57,58,59]. The system consists of the BL-aluminosilicate film adsorbed on Ru(0001) in a 5.392 Å × 9.339 Å × 27 Å super cell, which includes five layers of Ru atoms in the slab model, the bilayer aluminosilicate, and O atoms adsorbed at the BL/Ru surface. A kinetic energy cutoff of 800 eV was used and the Brillouin zone was sampled with an 8 × 4 × 1 mesh. The reaction pathways and energy barriers were calculated using the climbing image nudged elastic band method (CI-NEB) [60] implemented in VASP. The BL-aluminosilicate, chemisorbed O atoms, and top two layers of Ru atoms were allowed to relax until forces were smaller than 0.02 eV/Å in the structural optimization and smaller than 0.05 eV/Å in the CI-NEB calculations.

## 3. Results and Discussion

### 3.1. Kinetic Study of the Water Formation Reaction (WFR) at Constant Pressure (0.1 Torr H_2_) by X-ray Photoelectron Spectroscopy

**Figure 3:**
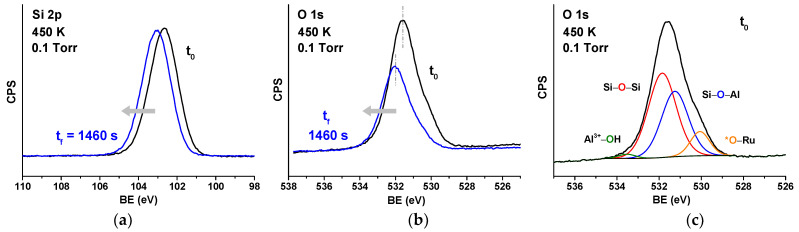
Si 2p (**a**) and O 1s (**b**) core level spectra before and after reaction at 450 K in 0.1 Torr of H_2_; (**c**) deconvolution of the O 1s core level spectrum before reaction.

For all the experiments shown in this work, the starting coverage of chemisorbed oxygen (*O) is estimated to be 0.375 ML, based on the O 1s peak area ratio between chemisorbed oxygen (*O) and the framework oxygen of the BL-aluminosilicate. The initial coverage was obtained by annealing the sample in an oxygen atmosphere (3 × 10^−6^ Torr) at 823 K for 30 min. The WFR was first studied in situ by AP-XPS at a H_2_ pressure of 0.1 Torr by acquiring alternatively the Si 2p and the O 1s core-level spectra as a function of time to follow the *O consumption evolution. This was done at four different temperatures: 380 K, 400 K, 420 K, and 450 K. Figure 3a and Figure 3b show respectively the XPS Si 2p and O 1s core-level spectra before (black line) and after (blue line) the WFR at the 2D-aluminosilicate/Ru(0001) interface at 450 K. In prior work, for the all-Si silica bilayer, the consumption of O 1s component corresponding to chemisorbed O was used to quantitatively follow the progress of the water formation reaction. In the current paper, the presence of Al in the framework complicated the reliable use of this method, given the additional component of framework oxygen bridging between Si and Al, and the fact that this O atom can also be in the hydroxylated (Figure 1c) and non-hydroxylated (Figure 1b) forms. The complexity of deconvoluting these components is shown in Figure 3c, where four peaks are used to deconvolute the O 1s region before the start of the reaction, at 450 K. This region can be deconvoluted into four peaks located at 533.5, 531.9, 531.3, and 530.0 eV, corresponding to O atoms in OH–Al^3+^ groups, Si–O–Si and Si–O–Al environments, and O chemisorbed on the Ru(0001) surface (*O), respectively. Given this, and the fact that four components can easily fit an elephant, we have chosen to use the shift of the Si 2p spectrum as a measure of the reaction progress. Note that in prior work [52], it has already been determined that the magnitude of the blueshift of Si 2p is proportional to the consumption of chemisorbed O, as the Ru–O dipoles are removed during the reaction. Figure S1 shows the plot of Si 2p shift vs. change in coverage that is used for reference.

**Figure 4:**
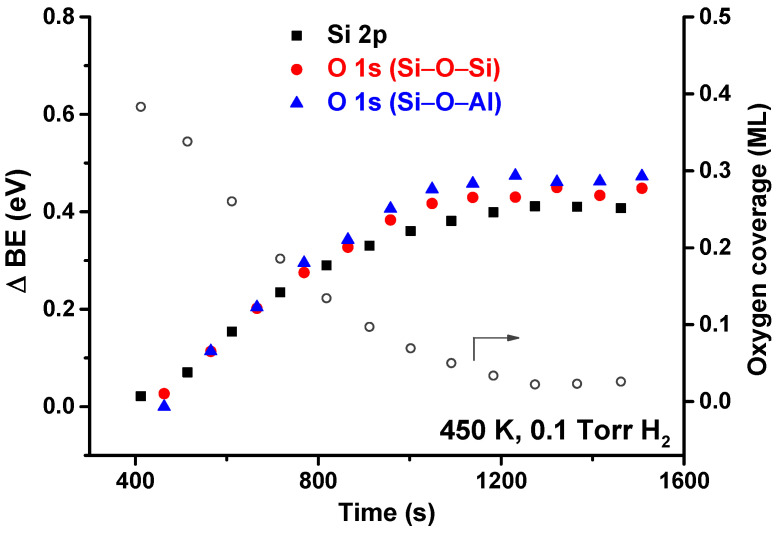
Core level shifts (solid symbols) for Si 2p and O 1s (Si–O–Si and Si–O–Al components) as a function of time at 450 K in 0.1 Torr of H_2_. The coverage of chemisorbed O (open circles) is also shown for comparison.

Figure 4 shows the shifts of Si 2p and O 1s (Si–O–Si and Si–O–Al) core levels (left axis, solid symbols) as a function of time at 450 K and 0.1 Torr of H_2_. The open circles (right axis) show the corresponding coverage of chemisorbed oxygen. As it was for the all-SiO_2_ bilayer, there is an induction period before the reaction starts taking place. Additionally, as the temperature stabilizes at the beginning of the reaction, the current in the filament that heats the sample takes a few minutes to stabilize. As the filament current is changing in this brief period, there are changes in the induced electric field, resulting in artifacts in the peak position. For this reason, we have chosen to discard these initial data points, and the plot starts at 400 s. The entire plot for all temperatures is included in the supporting information.

**Figure 5:**
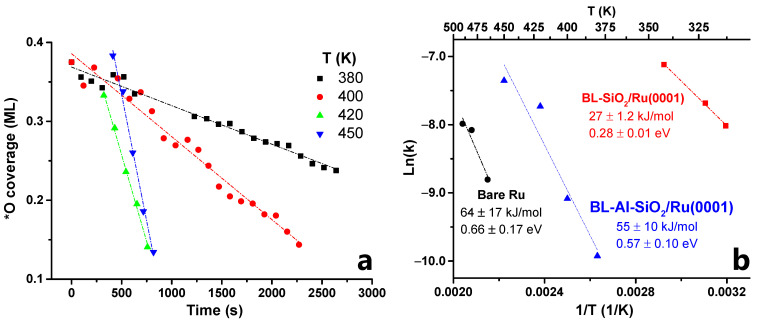
(**a**) Plot of oxygen coverage vs. time at 380, 400, 420, and 450 K; (**b**) Arrhenius plots for WFR at the BL-aluminosilicate/Ru(0001) interface (this work, blue triangles), compared to similar prior work on bare Ru(0001) (black circles) and BL-silica/Ru(0001) (red squares).

In Figure 5a, we plot the linear part of the coverage (after the induction period) vs. time, in order to obtain the initial rate of reaction at four different temperatures, namely 380, 400, 420, and 450 K. This temperature range was chosen so that obtained rates of consumption of chemisorbed oxygen could be tracked by ambient-pressure XPS considering the constraints of our time resolution. This rate was then used to obtain the Arrhenius plot shown in Figure 5b (blue triangles). An apparent activation energy of 55 kJ/mol was obtained. This was much higher than the case of the all-Si bilayer, but lower than the case of bare Ru. The Arrhenius plots for these cases (reproduced from [52]) are also included in Figure 5b for comparison.

### 3.2. Density Functional Theory (DFT) Calculations

DFT calculations revealed that the rate-limiting step of the water formation reaction (WFR) is the formation of *OH on the Ru(0001) surface via the first hydrogen addition step (*H + *O ↔ *OH) [46,52]. Bilayer silica films (Figure 1a) create a large desorption barrier that trap water molecules at the interface (*d*_Ru–O_ = 3.85 Å) and activate an alternative disproportionation reaction pathway (*H_2_O + *O ↔ 2 *OH; TS2′ in Figure 2) to form *OH groups, with a barrier 0.25 eV lower than the first hydrogen addition step [52]. The structure of aluminosilicate film is similar to that of the bilayer silica film, and thus it is expected to also trap water molecules at the interface. Here, we perform DFT calculations to study both reaction pathways to determine the energy barriers for WFR at the aluminosilicate/Ru interface.

Figure 1b shows the super cell of the bilayer aluminosilicate/3O/Ru(0001) system including two aluminosilicate nano-cages, eight surface Ru atoms, and three O atoms adsorbed on of the Ru surface. The O coverage corresponds to 0.375 monolayer (ML) in the experiment. The Al concentration in the BL-aluminosilicate is 25%: two Al atoms are included in a unit cell [(Al_0.25_Si_0.75_O_2_)_8_]. The substitution of Si with Al results in an [AlO_4_]^−^ center that attracts an extra electron to saturate one O to form four Al–O bonds. The negative charge on [AlO_4_]^−^ is locally compensated by a proton or another cation. Our previous studies have shown that this charge compensation can also be supplied by the Ru substrate [34]. Upon H_2_ adsorption, we found that the adsorption energy of two H atoms is much larger in magnitude at the bottom layer of the BL-aluminosilicate than on the Ru surface, indicating that charge compensation from the H atoms is more stable than the Ru substrate. Therefore, our studies on the water formation reaction (WFR) start from a new substrate [(Al_0.25_Si_0.75_O_2_)_8_–2H/3O/Ru (Figure 1c,d)] where two H atoms are bonded to two O atoms in the bottom layer of the BL-aluminosilicate. Due to the adsorption of H, the interface space (*d*_Ru–O_ = 3.55 Å) is much larger than the aluminosilicate/3O/Ru system (*d*_Ru–O_ = 2.47 Å in Figure 1b).

**Figure 6:**
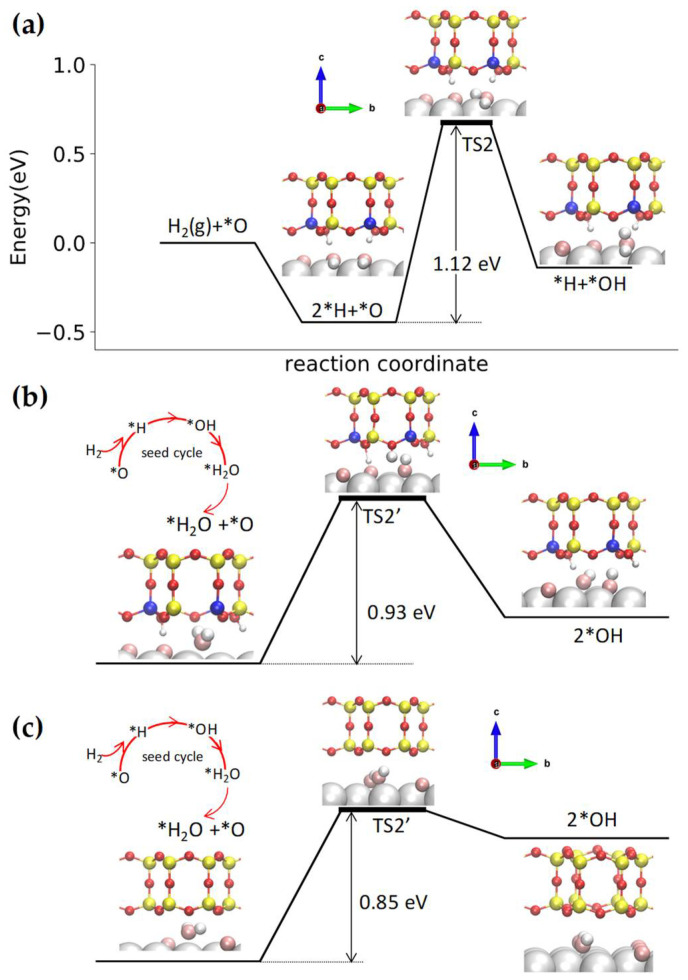
Potential energy diagram for the WFR at the BL-aluminosilicate/Ru(0001) interface via (**a**) first hydrogen addition step (*H + *O ↔ *OH) and (**b**) disproportionation pathway (*H_2_O + *O ↔ 2 *OH); (**c**) Potential energy diagram for the disproportionation pathway (*H_2_O + *O ↔ 2 *OH) at the silica/Ru(0001) interface. Color code: Ru (silver), Si (yellow), Al (blue), H adsorbed on aluminosilicate (small white), O in aluminosilicate (red), *O chemisorbed on Ru (pink), and *H adsorbed at the aluminosilicate/Ru(0001) interface that react with *O (large white).

Figure 6a shows the reaction pathway for the first hydrogen addition reaction (*H + *O ↔ *OH), where a *H atom migrates to bond to an *O atom on Ru. The activation energy is 1.12 eV, which is close to that of the bare Ru and silica/Ru interface [52]. The water molecules formed from the initial first hydrogen addition reaction can be stabilized by the BL-aluminosilicate film, which activates the disproportionation pathway. The initial state of the disproportionation pathway involves a water molecule adsorbed at the interface. Figure 6b shows that one of the *H atoms of *H_2_O migrates to combine with a nearby *O atom (*H_2_O + *O ↔ 2 *OH) with an activation energy of 0.93 eV, which is lower than the hydrogen addition reaction.

To compare with the disproportionation reaction at the silica/Ru interface, we also show this pathway in Figure 6c. The activation energy of *H_2_O + *O ↔ 2 *OH at the aluminosilicate/Ru interface (0.93 eV) is slightly higher than the silica/Ru interface (0.85 eV in Figure 6c) [52]. Moreover, the disproportionation reaction at the silica/Ru interface only involves *H and *O atoms migrating on Ru. In the aluminosilicate case, one *H atom dissociates from the water molecule, and its migration to the nearby *O atom is facilitated by a framework O atom bridging between Si and Al, as seen in the transition state in Figure 6b.

### 3.3. Reaction Order with Respect to H_2_

**Figure 7:**
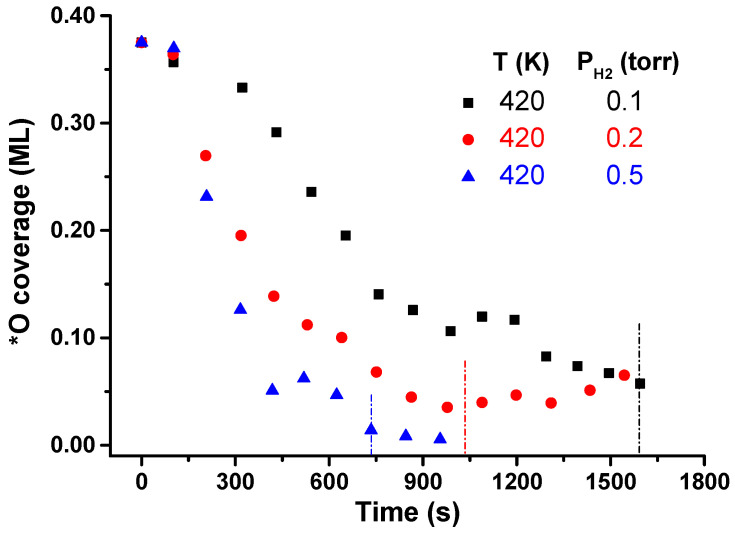
WFR reaction evolution at 420 K and variable pressure conditions (0.1–0.5 Torr H_2_). Vertical lines indicate the endpoint of the reaction at each working pressure.

Another interesting feature to analyze in the WFR under interfacial confinement is the impact of H_2_ pressure during the catalytic process. In Section 3.1, 0.1 Torr H_2_ was set at a constant pressure, and the temperature was varied from 380 to 450 K. In this second set of experiments, the pre-activation of the sample was identical (3 × 10^−6^ Torr O_2_ at 823 K, 30 min). The initial O coverages were also 0.375 ML. Based on the results obtained at variable temperature, 420 K was chosen to carry out the experiments, and three H_2_ pressures were used, namely: 0.1, 0.2, and 0.5 Torr. Figure 7 shows the θ_*__O_ evolution for these three pressures. For 0.1 Torr H_2_, the endpoint of the reaction is reached in ~27 min. Increasing the pressure increases the reaction rate, reaching the final coverage at approximately 17 min at 0.2 Torr and 12 min at 0.5 Torr H_2_.

**Figure 8:**
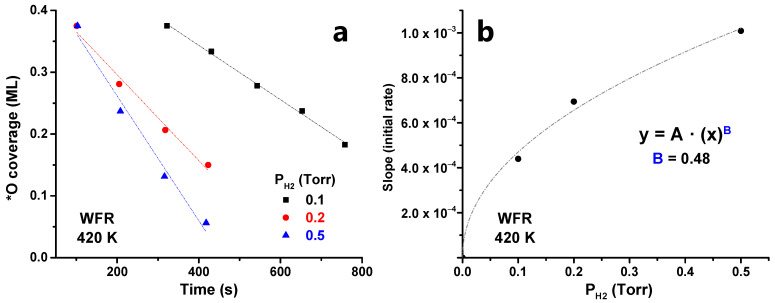
(**a**) Initial WFR reaction evolution at different temperatures (380–450 K) at 0.1 Torr H_2_ and (**b**) dependence between the initial rate and the H_2_ pressure.

Examining the initial catalytic evolution, we observe that θ_*__O_ decreases linearly with time at all pressure ranges after the induction period. This linear region is shown in Figure 8a. Then, the kinetic constant (k) can be calculated by assuming zero-order kinetics with respect to θ_*__O_, as done in Section 3.1. By plotting the value of the kinetic constant (k) vs. H_2_ pressure, an exponential fitting can be proposed, obtaining a reaction order of ~0.5 with respect to H_2_ (Figure 8b). Note that this reaction order of 0.5 is based on only three data points and, while it provided a reasonable approximation, further experiments would be needed to obtain a more accurate value.

## 4. Conclusions

In this work, we studied the water formation reaction at the confined interface between an alumino-silicate bilayer and Ru(0001) surface (i.e., Al_0.25_Si_0.75_O_2_/Ru(0001)). The system can be thought of as a nanoreactor. The reaction kinetics were followed by synchrotron-based ambient pressure X-ray photoelectron spectroscopy (AP-XPS), complemented by DFT calculations.

First, a catalytic study on the model system at constant H_2_ pressure (0.1 Torr) and variable temperature (380–450 K) was performed. In all cases, the initial *O-coverage was 0.375 ML. The temporal evolution of chemisorbed oxygen on the Ru(0001) surface during the reaction was monitored by AP-XPS at each temperature. These data were used to obtain rate constants from the initial reaction rates to produce an Arrhenius plot. An apparent activation energy of 55 kJ/mol was determined. This value is similar to that obtained for the bare Ru (64 kJ/mol) and surprisingly higher than the E^app^ value recently reported by our group in the pure BL-SiO_2_/Ru(0001) (i.e., 27 kJ/mol).

The presence of aluminum in the doped-bilayer introduces negative charges in the framework that are likely compensated by a proton bound to a bridging O (Si–O–Al). Therefore, the substrate used to carry out the theoretical calculations was [(H_0.25_Al_0.25_Si_0.75_O_2_)_8_/3O/Ru], exhibiting a slightly smaller interfacial space (*d*_Ru–O_ = 3.55 Å) than its pure silicate (*d*_Ru–O_ = 3.85 Å) counterpart. While the confinement at such an interface favors the disproportionation pathway, as was the case for bilayer silica (at the conditions used in our work), the activation energy for the hydrogen is higher than in the pure silica (0.93 eV for the BL-Al–SiO_2_ versus 0.85 eV for the pure BL-SiO_2_). This agrees with the experimentally obtained apparent activation energy lower than that of bare Ru(0001) but higher than that of bilayer silica.

Finally, the impact of H_2_ pressure in the WFR was evaluated. The H_0.25_Al_0.25_Si_0.75_O_2_)_8_/3O/Ru was kept at a temperature of 420 K, while the kinetic experiment was run at different pressures. This allowed us to determine a reaction order with respect to H_2_ of 0.5.

## Data Availability

Data is included in the article and supporting information in the form of graphs and tables. Raw data can be obtained from the corresponding authors upon request.

## Supplementary Materials

The following are available online at https://www.mdpi.com/article/10.3390/nano12020183/s1, Figure S1: *O coverage vs. BE shift of the Si 2p core level. This obtained relation was used to determine coverage in our experiments based on the measured BE shift; Figure S2: Temporal evolution of chemisorbed O-coverage at 380 K, 400 K, 420 K, and 450 K.
