# Supplementary material for: Water Formation Reaction under Interfacial Confinement: Al0.25Si0.75O2 on O-Ru(0001)
Source: Nanomaterials (Basel). 2022 Jan 6;12(2):183. doi: 10.3390/nano12020183 (PMC8779344; doi:10.3390/nano12020183)
Supplement: Supplementary file 1 [file nanomaterials-12-00183-s001.zip › nanomaterials-1512489-supplementary.pdf]

nanomaterials

MDPI

Supporting Information

Water Formation Reaction under Interfacial Confinement: $Al_{0.25}Si_{0.75}O_2$ on O-Ru(0001)

Jorge Cored [1,2], Mengen Wang [2,3], Nusnin Akter [2,3], Zubin Darbari [2,3], Yixin Xu [2,3], Burcu Karagoz [2], Iradwikanari Waluyo [4], Adrian Hunt [4], Dario Stacchiola [2], Ashley Rose Head [2], Patricia Concepcion [1], Deyu Lu [2,*] and Jorge Anibal Boscoboinik [2,*]

[1] Instituto de Tecnología Química, Universitat Politècnica de València-Consejo Superior de Investigaciones Científicas (UPV-CSIC), Avenida de los Naranjos s/n, 46022 Valencia, Spain; jorcoban@upvnet.upv.es (J.C.); pconcepc@upvnet.upv.es (P.C.)
[2] Center for Functional Nanomaterials, Brookhaven National Laboratory, Upton, NY 11973, USA; mwang.mse@gmail.com (M.W.); nusninakter@gmail.com (N.A.); zubin.darbari@stonybrook.edu (Z.D.); yixin.xu@stonybrook.edu (Y.X.); bkaragoz@bnl.gov (B.K.); djs@bnl.gov (D.S.); ahead@bnl.gov (A.R.H.)
[3] Materials Science and Chemical Engineering Department, Stony Brook University, Stony Brook, NY 11790, USA
[4] National Synchrotron Light Source II, Brookhaven National Laboratory, Upton, NY 11973, USA; iwaluyo@bnl.gov (I.W.); adhunt@bnl.gov (A.H.)
* Correspondence: dlu@bnl.gov (D.L.); jboscoboinik@bnl.gov (J.A.B.)

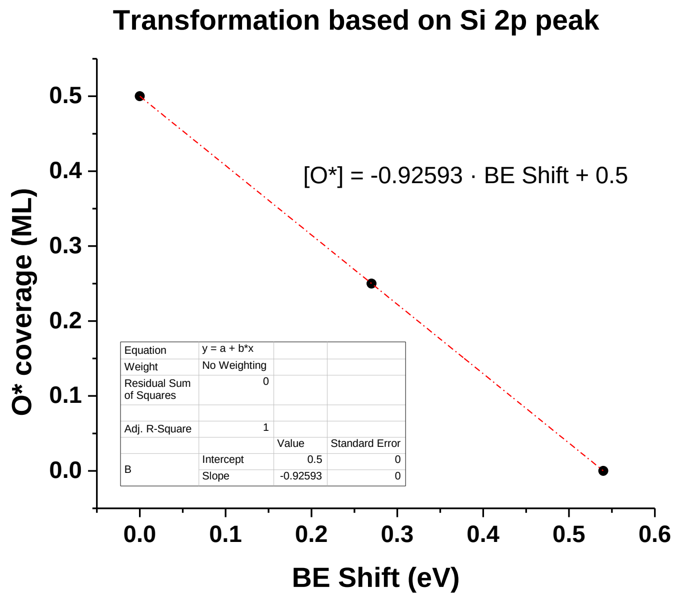

Figure S1. O* coverage vs BE shift of the Si 2p core level. This obtained relation was used to determine coverage in our experiments based on the measured BE shift.

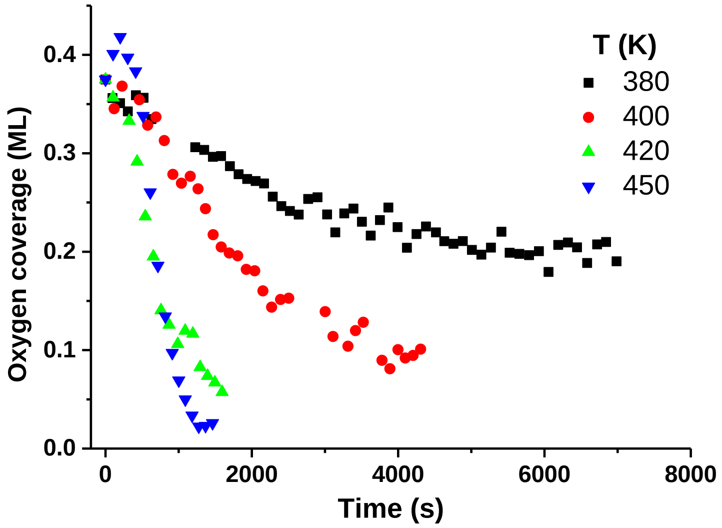

Figure S2. Temporal evolution of chemisorbed O-coverage at 380 K, 400 K, 420 K, and 450 K.
